# Comprehensive characterization of ubiquitinome of human colorectal cancer and identification of potential survival-related ubiquitination

**DOI:** 10.1186/s12967-022-03645-8

**Published:** 2022-10-02

**Authors:** Wei Zhang, Yan Yang, Liewen Lin, Jingquan He, Jingjing Dong, Bin Yan, Wanxia Cai, Yumei Chen, Lianghong Yin, Donge Tang, Fanna Liu, Yong Dai

**Affiliations:** 1 The First Affiliated Hospital, Jinan University, 613 W. Huangpu Avenue, Guangzhou, Guangdong 510632 China; 2 Department of Nephrology, Institute of Nephrology and Blood Purification, The First Affiliated Hospital of Jinan University, Jinan University, Guangzhou, Guangdong 510632 China; 3 Department of Clinical Medical Research Center, Guangdong Provincial Engineering Research Center of Autoimmune Disease Precision Medicine The Second Clinical Medical College, Jinan University (Shenzhen People’s Hospital), Shenzhen, Guangdong 518020 China

**Keywords:** Ubiquitinome, Multi-omics study, Colorectal cancer, FOCAD, DOCK2

## Abstract

**Background:**

According to the Global Cancer Statistics in 2020, the incidence and mortality of colorectal cancer (CRC) rank third and second among all tumors. The disturbance of ubiquitination plays an important role in the initiation and development of CRC, but the ubiquitinome of CRC cells and the survival-relevant ubiquitination are poorly understood.

**Methods:**

The ubiquitinome of CRC patients (n = 6) was characterized using our own data sets of proteomic and ubiquitin-proteomic examinations. Then, the probable survival-relevant ubiquitination was searched based on the analyses of data sets from public databases.

**Results:**

For the ubiquitinomic examination, we identified 1690 quantifiable sites and 870 quantifiable proteins. We found that the highly-ubiquitinated proteins (n ≥ 10) were specifically involved in the biological processes such as G-protein coupling, glycoprotein coupling, and antigen presentation. Also, we depicted five motif sequences frequently recognized by ubiquitin. Subsequently, we revealed that the ubiquitination content of 1172 proteins were up-regulated and 1700 proteins were down-regulated in CRC cells versus normal adjacent cells. We demonstrated that the differentially ubiquitinated proteins were relevant to the pathways including metabolism, immune regulation, and telomere maintenance. Then, integrated with the proteomic datasets from the Clinical Proteomic Tumor Analysis Consortium (CPTAC) (n = 98), we revealed that the increased ubiquitination of FOCAD at Lys583 and Lys587 was potentially associated with patient survival. Finally, we depicted the mutation map of FOCAD and elucidated its potential functions on RNA localization and translation in CRC.

**Conclusions:**

The findings of this study described the ubiquitinome of CRC cells and identified abnormal ubiquitination(s) potentially affecting the patient survival, thereby offering new probable opportunities for clinical treatment.

**Supplementary Information:**

The online version contains supplementary material available at 10.1186/s12967-022-03645-8.

## Background

According to the Global Cancer Statistics in 2020, the incidence and mortality of colorectal cancer respectively rank third and second [1, 2]. Although the emergence of targeted and immune therapies in recent years has improved the five-year survival rates of CRC patients [3–5], the advanced and metastatic tumors of most patients still fail to receive remission [6].

Ubiquitination is a post-translational modification, which mainly regulates protein functions via degrading and transporting. Nowadays, more and more evidence has demonstrated that abnormal protein ubiquitination plays an essential role in carcinogenesis, influencing a variety of processes in tumor cells, such as cell cycle, proliferation, differentiation, and apoptosis [7]. In 2020, Yuan X et al. uncovered the ubiquitination events in metastatic colonic tissues versus primary colonic tissues [8]. However, the ubiquitinome of human CRC cells versus normal cells and the survival-relevant ubiquitination is barely known.

FOCAD encodes a protein named Focadhesin [9]. Several studies have declaimed that FOCAD may be associated with the functions of cell cycle, proliferation, apoptosis, and cell adhesion [10]. Previous studies have shown that FOCAD is potentially a tumor suppressor in CRC, lung cancer, and astrocytoma [11]. Patients with germline deletions of FOCAD are more likely to develop intestinal polyps and tumors [12]. Besides, the functions of FOCAD in tumorigenesis are potentially relevant to the regulation of cystine deprivation-induced ferroptosis [13]. In contrast, the association between the FOCAD expression and patient outcomes and its post-translational modification in CRC cells has not been studied.

In this study, we collected the cancerous and para-cancerous tissues from six CRC patients and investigated the ubiquitinome of CRC cells versus normal cells using liquid chromatography-mass spectrometry. Subsequently, we calculated all ubiquitinated proteins and explored their probable related biological processes. We also analyzed the sequences highly recognized by ubiquitin in CRC cells. Next, we investigated the proteins whose expression content and ubiquitination content were opposite and explored the pathways in which they potentially involved. Ultimately, we screened for the probably functional ubiquitin-modifying proteins.

## Methods

### Patients

Approved by the Ethics Committee of Shenzhen People's Hospital, the cancerous intestinal mucosa tissues from six colon adenocarcinoma patients were obtained from Shenzhen People’s Hospital. We fully explained the purpose and experimental procedures to each patient. They all volunteered to participate in the program and signed the informed consent. All issues of CRC patients were collected with reference to a reported experimental method [14]. Tumor tissues were taken from the colon segment, and the normal adjacent colon mucosa was removed within 5 cm of the tumors. All tissues were collected within 0.5 h after surgery, frozen in liquid nitrogen immediately for more than three hours, and stored at − 80 °C.

### Trypsin digestion

Trichloroacetic acid (TCA) was slowly added in each sample, followed centrifugation at 4500 g at 4 °C for 5 min, then the supernatant was discarded. The remaining debris was washed with pre-cooled acetone once, dried in the air, and dissolved with 200 mM tetraethyl ammonium bromide (TEAB). Subsequently, the trypsin was added to the remaining debris and reacted with the sample overnight. After incubating with the dithiothreitol at 56 °C for 30 min, the remaining debris was added with iodoacetamide (IAA) and then incubated at room temperature for 15 min in the dark.

### Liquid chromatography mass spectrometry (LC–MS)/MS analyses

The LC–MS/MS analysis was performed following a standard protocol [15]. The peptides were dissolved with mobile phase A (0.1% formic acid, 2% acetonitrile), then the mixtures were separated using the Nano Elute ultra-high performance liquid system. The gradient of mobile phase B (containing 0.1% formic acid and 100% acetonitrile) was set as follows: 0–44 min, 6–22% mobile phase B; 44–56 min, 22–30% mobile phase B; 56–58 min, 30–80% mobile phase B; 58–60 min, 80% mobile phase B. The flow rate was set as 450.00 nl/min. The separated peptides were then injected into the capillary ion source for ionization and analyzed using the times-TOF Pro mass spectrometer. The ion source voltage was set into 2.0 kV. The scanning range of secondary MS was set into 100–1700 m/z. Parallel accumulation serial fragmentation (PASEF) mode was used for data acquisition. After the first mass spectrum, the secondary mass spectrum was collected ten times in the PASEF mode, and the dynamic exclusion time of tandem MS scanning was set into 30 s.

### Database search

The secondary MS data were searched against the Homo sapiens 9606 (20,366 sequences) with the common contamination database using the Maxquant search engine (1.6.6.0). The reverse database was added to calculate the false discovery rate (FDR) caused by random matching. All parameters were set as follows: the FDR and peptide spectrum matches (PSM): 1%; Enzyme digestion: trypsin/P; Number of missed sites: 4; Minimum length: 7; Maximum modification number: 5; Mass error tolerance of primary parent ion and the main search: 20 ppm in the first search; Fragment ions: 0.02 Da; Fixed modification: cysteine alkylation carbamidomethyl; Variable modification: acetyl (protein N-term), oxidation (m); glygly (k).

### Protein annotation and functional enrichment

Gene Ontology (GO) annotation was performed using the UniProt-GOA database (http://www.ebi.ac.uk/GOA). Kyoto Encyclopedia of Genes and Genomes (KEGG) database (http://www.genome.jp/kegg/) was utilized to annotate the differentially ubiquitinated proteins into pathways. The mRNA expression was analyzed using the datasets from Gene Expression Profiling Interactive Analysis (GEPIA) database (https://www.ncbi.nlm.nih.gov/geo/). The protein expression was investigated using the datasets from The National Cancer Institute’s Clinical Proteomic Tumor Analysis Consortium (CPTAC) database (https://portal.gdc.cancer.gov/). The immunohistochemical detection was obtained from The Human Protein Altas (HPA) database (www.proteinatlas.org).

### Statistical analysis

The ratio of the relative quantitative value of each protein in the tissue was considered as a fold change (FC). The calculation formula was as follows: FC A/B, k = RAk / RBk (‘‘A’’/‘‘B’’ represented different tissues, ‘‘R’’ represented the relative quantitative value of the protein, and ‘‘k’’ represented the protein). Based on the above analysis of differences, the change of differential expression more than 1.5 was regarded as the threshold of significant up-regulation, and that less than 1/1.5 was regarded as the threshold of significant down-regulation. Fisher’s exact tests were used to evaluate the significance of enrichment results. A P value of less than 0.05 was considered being statistically significant.

## Results

### Identification of the ubiquitinome of CRC cells and normal adjacent cells

To characterize the ubiquitinome of CRC patients, we collected the cancerous and para-cancerous tissues from six CRC patients and performed proteomic and ubiquitinomic analyses on them. The clinical information of the six CRC patients was summarized in Table 1, including age, type, gender, tumor/node/metastasis (TNM) stage, and chemotherapy. As shown in Additional file 1: Figure S1, most of the peptide lengths were distributed in 7–20 amino acids, which met the quality control requirements. For proteomic examination, we identified 86,259 matched spectrums, 43,073 peptides, 41,367 unique peptides, 6080 identified proteins, and 4712 quantifiable proteins. For ubiquitinomic examination, we identified 16,615 matched spectrums, 9295 peptides, 5537 identified sites, 5494 modified peptides, 2410 identified proteins, 1690 quantifiable sites, and 870 quantifiable proteins (Additional file 1: Figure S2, Additional file 2: Table S1,S2).

**Table 1:** The characteristics of the six CRC patients participating in our study

Characteristics	I–II (%)		III–IV (%)	
Age				
≤ 65	0	(0.0)	2	(33.3)
> 65	2	(33.3)	2	(33.4)
Type				
Adenocarcinoma	2	(33.3)	4	(66.7)
Gender				
Male	1	(16.7)	3	(50.0)
Female	1	(16.7)	1	(16.6)
T stage				
T1	1	(16.7)	0	(0.0)
T2	0	(0.0)	0	(0.0)
T3	0	(0.0)	2	(33.3)
T4	1	(16.7)	2	(33.3)
N stage				
N0	2	(33.3)	0	(0.0)
N1	0	(0.0)	2	(33.3)
N2	0	(0.0)	2	(33.4)
M stage				
M0	2	(33.3)	2	(33.3)
M1	0	(0.0)	2	(33.4)
Locations				
Ascending colon	1	(16.7)	1	(16.6)
Sigmoid colon	1	(16.7)	3	(50.0)
Appendix colon	0	(0.0)	0	(0.0)
Therapy				
Yes	0	(0.0)	0	(0.0)
No	2	(33.3)	4	(66.7)

**Fig. 1:**
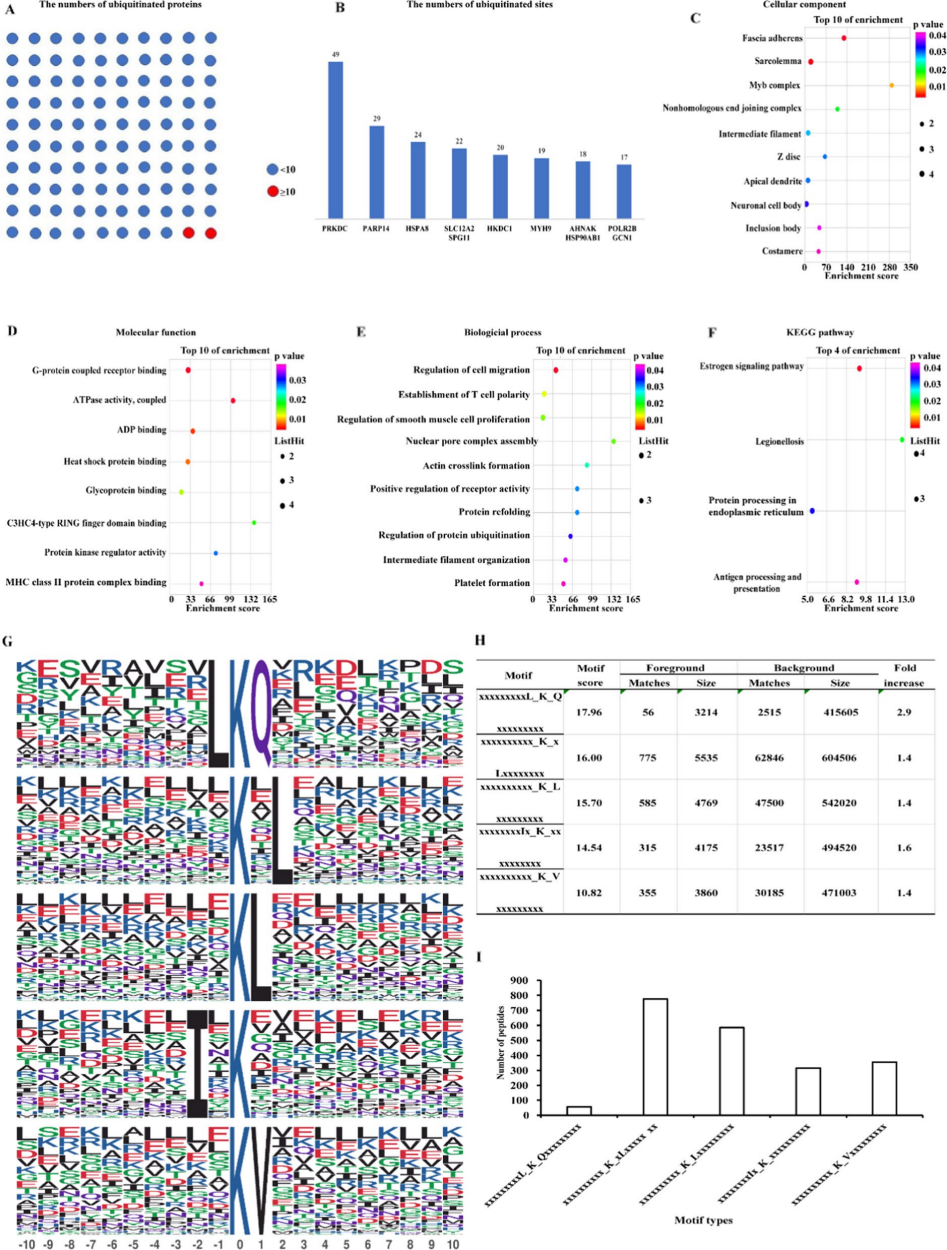
The characterization of ubiquitinome of CRC cells and the identification of ubiquitin-binding motifs in colonic cells. **A** The numbers of proteins with more than 10 ubiquitin-modified sites or with less than 10 ubiquitin-modified sites (each circle represented 20 proteins). **B** Highly ubiquitinated proteins (n ≥ 10) and the numbers of ubiquitin-modified sites. (**C**–**E**) GO analyses uncovered the specific modules that the highly-ubiquitinated proteins enriched in. **F** KEGG analyses uncovered the specific modules that the highly-ubiquitinated proteins enriched in. **G** The top five ubiquitin-binding motifs. The height of each letter corresponded to the frequency of this amino acid residue in the position. The central K refers to the ubiquitinated Lys. **H** The motif scores of the top five ubiquitination motifs. **I** The numbers of peptides covered by the motifs

In eukaryotes, ubiquitination is essential for many biological processes, including cell survival and differentiation, as well as for innate and adaptive immunity. The analysis of the biological processes and pathways in which the highly-ubiquitinated proteins are involved may provide a preliminary exploration of the role of ubiquitination. Therefore, we enriched all the ubiquitinated proteins and found that 98.3% of total proteins (2409 proteins) harbored less than 10 ubiquitination modifications and 1.7% (42 proteins) harbored 10 or more ubiquitination modifications (Fig. 1A, Additional file 2: Table S3), such as PRKDC, PARP14, SLC12A2, SPG10, HKDC1, MYH9, HSPA8, AHNAK, HSP90AB1, POLR2B, and GCN1. Among the highly-ubiquitinated proteins (n ≥ 10), PRKDC was the most modified one among all proteins, which had 49 ubiquitination sites in total (Fig. 1B). To better understand the functions of high-ubiquitinated proteins, we performed GO and KEGG analyses of the high-ubiquitinated proteins and the low-ubiquitinated proteins, respectively. After removing the same modules shared with low-ubiquitinated proteins, we found that the high-ubiquitinated proteins were especially enriched in modules of G-protein-coupled receptors, ATPase activity, immunity, and cytoskeleton (Fig. 1C–F).

### Motif analysis disclosed the sequences more inclined to be ubiquitinated

Subsequently, we searched for the motifs frequently modified by the ubiquitin in normal and cancerous colonic tissues using Motif-X (Fig. 1G). As a result, 20 motifs were screened out Additional file 2: Table S4) and the top five motifs were LKQ, KxL, KL, IxK, and KV (Fig. 1H). These five motifs were distributed in 56, 775, 585, 315, and 355 unique peptides, respectively (Fig. 1I).

### The proteins with differentially expressed ubiquitination were potentially associated with metabolism, immune regulation, and telomere maintaining

**Fig. 2:**
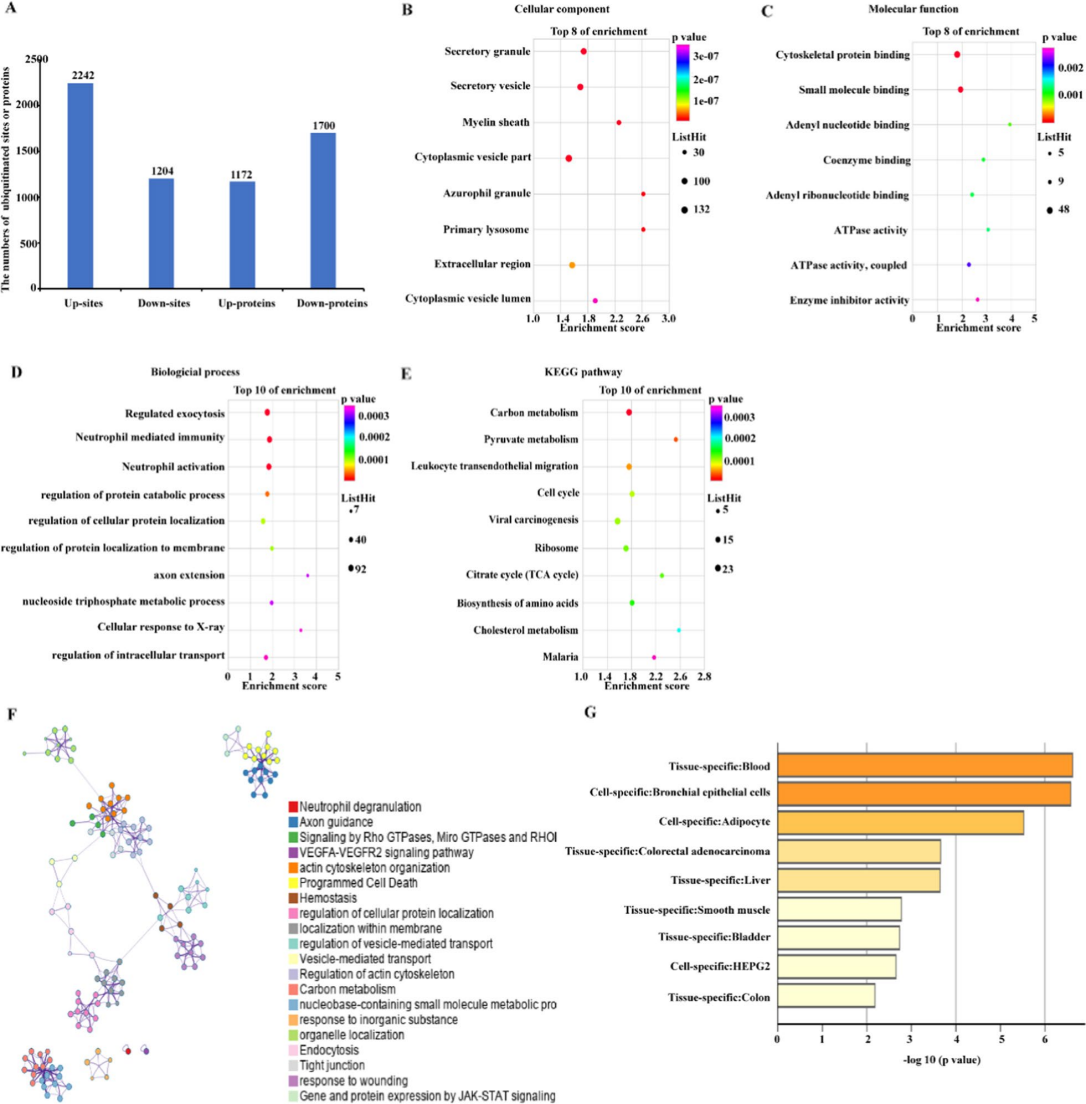
The functions of the differentially expressed ubiquitination of CRC cells versus normal cells. **A** The numbers of differentially ubiquitinated proteins and sites of CRC cells versus normal cells (fold change > 1.5, and the ubiquitination solely detected in cancer samples or normal adjacent samples were also included). (**B**–**D**) The GO enrichment of all differentially ubiquitinated proteins. **E** The KEGG enrichment of all differentially ubiquitinated proteins. **F** Functional enrichment of secretory proteins having differentially-expressed ubiquitination, as analyzed using the Metascape database. **G** The cellular sources of secretory proteins having differentially-expressed ubiquitination, as analyzed using the PaGenBase database

To explore the functions of disturbed ubiquitination in CRC, we analyzed the differentially expressed ubiquitination in CRC cells versus normal cells. As a result, the ubiquitination contents of 1172 proteins were up-regulated, and the ubiquitination contents of 1700 proteins were down-regulated. Meanwhile, the ubiquitination contents of 2242 sites were up-regulated, and the ubiquitination contents of 1204 sites were down-regulated (fold change > 1.5, the ubiquitination solely detected in cancer samples or normal adjacent samples were also included) (Fig. 2A, Additional file 2: Table S5, S6). Subsequently, we performed GO and KEGG enrichment analyses of these different ubiquitinations, and found that the proteins with altered ubiquitination were mainly enriched in secretory vesicle, myelin sheath, and cytoplasmic vesicle part, etc. (Fig. 2B). This result suggested that secreted proteins were more likely to be modified by ubiquitin, and thus ubiquitination might play an important role in protein transporting and secretion. Meanwhile, the ubiquitination-altered proteins were also enriched in processes including cytoskeletal protein binding, small molecule binding, adenyl nucleotide binding, and coenzyme binding, etc. (Fig. 2C), and involved in the purine nucleotide metabolic process, nucleotide metabolic process, neutrophil mediated immunity, neutrophil activation, etc. (Fig. 2D). This result revealed that the differentially expressed ubiquitination in CRC cells versus normal cells might be associated with metabolic and immune regulation. Next, the KEGG enrichment analysis uncovered that the differentially expressed ubiquitination was enriched in pathways including carbon metabolism, pyruvate metabolism, leukocyte transendothelial migration, cell cycle, viral carcinogenesis, ribosomes, citrate cycle, etc. (Fig. 2E). As observed in Fig. 2B, the proteins harboring differential ubiquitination were mostly secretory proteins. To further explore the functions of these proteins, we performed functional analysis of these secretory proteins (Additional file 2: Table S7) using the Metascape database, and the results showed that the functions of secretory proteins were closely related to neutrophil degranulation and JAK-STAT pathways. The JAK-STAT pathway is closely related to inflammation [16], so this result further confirmed a probably new function of ubiquitination modification in the immune responses (Fig. 2F). At the same time, we also analyzed the cellular sources of secretory proteins through PaGenBase database, and found that these proteins were more located in blood cells (Fig. 2G), which further revealed that ubiquitination modification might play a role in the immune responses.

**Fig. 3:**
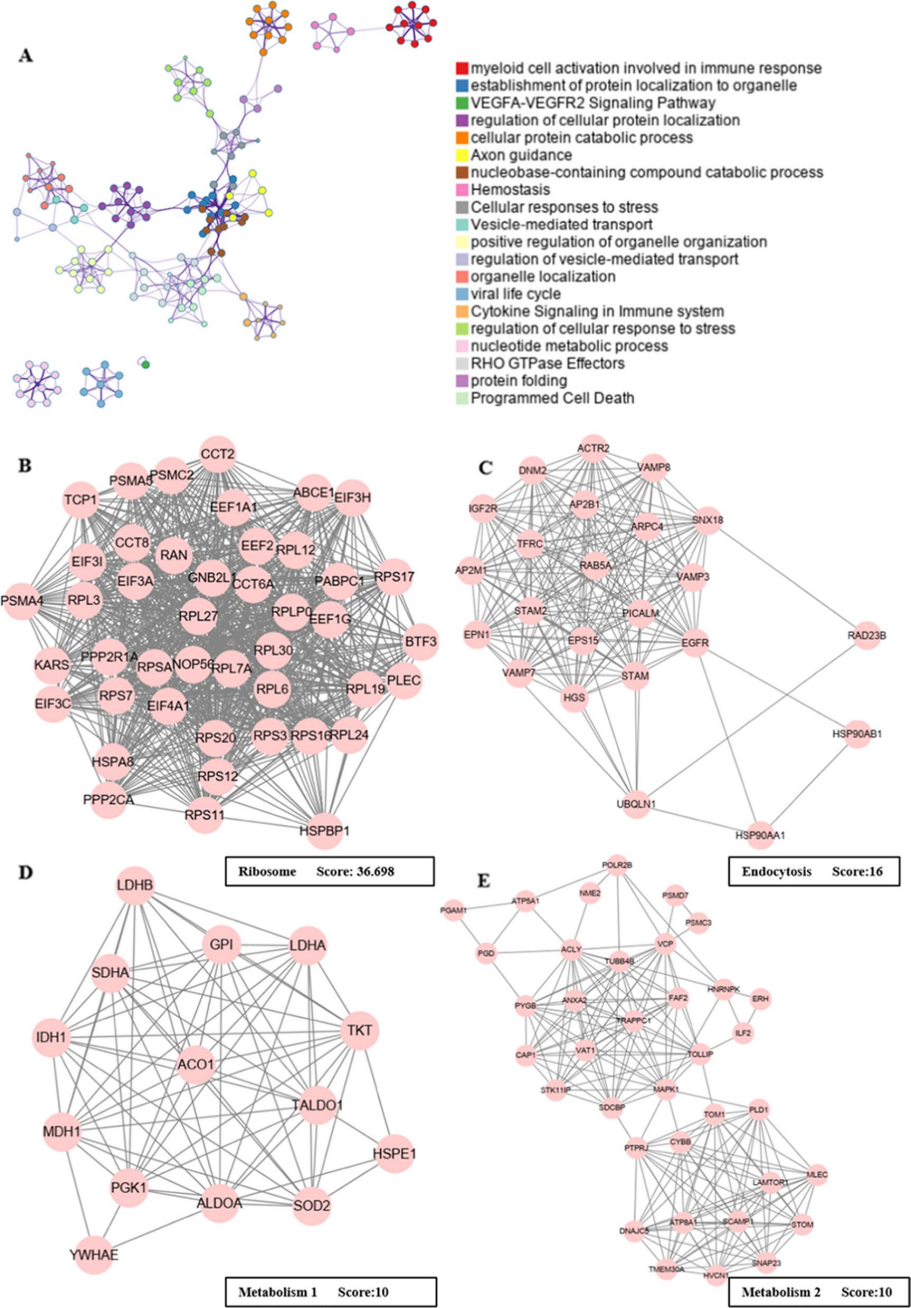
The functional network of all differentially ubiquitinated proteins and the closely-tied protein groups. **A** The functional network of all differentially ubiquitinated proteins. (**B–E**) The four protein groups within the network

To further analyze the functional network of differentially-ubiquitinated proteins, we annotated the genes using the Metascape database (Fig. 3A). Consistently, we enriched the pathways including myeloid cell activation involved in immune response and cytokine signaling in the immune system, highlighting the role of ubiquitination in regulating the immune microenvironment of CRC patients (Fig. 3A). Subsequently, we searched for the functional modules within the network using MCODE. As a result, modules named Ribosome, Endocytosis, Metabolism 1, and Metabolism 2 were discovered (Fig. 3B–E).

### The increased ubiquitination of FOCAD at Lys583 and Lys587 were potentially associated with the overall survival rates (OS) of CRC patients

Numerous previous studies have revealed that ubiquitination has a variety of functions, such as protein degradation, receptor internalization [17], protein assembly [18], transporting [19], signal transduction [20], and enzymatic activity modulation [21]. In the study, we focused on the function of protein degradation, and thus we searched for the differentially expressed proteins simultaneously with the opposite changes of ubiquitination. As a result, 53 proteins had up-regulated expression simultaneously with down-regulated ubiquitination, and 116 proteins had down-regulated expression simultaneously with up-regulated ubiquitination (169 in total) (Additional file 1: Figure S3, Additional file 2: Table S8). Then, the expression of the above 169 proteins were verified using the data set from CPTAC, and 59 proteins were confirmed (7 proteins were up-regulated and 52 proteins were down-regulated) (Additional file 1: Figure S3, Additional file 2: Table S9, S10). Subsequently, to screen out the functional ubiquitination potentially associated with CRC progression, we performed survival analyses using the proteomic datasets and the clinical information from the CPTAC database (n = 98). We found that 101 proteins were probably associated with the OS of CRC patients (Additional file 2: Table S11). We overlapped the 59 proteins selected in Additional file 1: Figure S3, and the 101 OS-relevant proteins and obtained two proteins ultimately, including FOCAD and DOCK2.

**Fig. 4:**
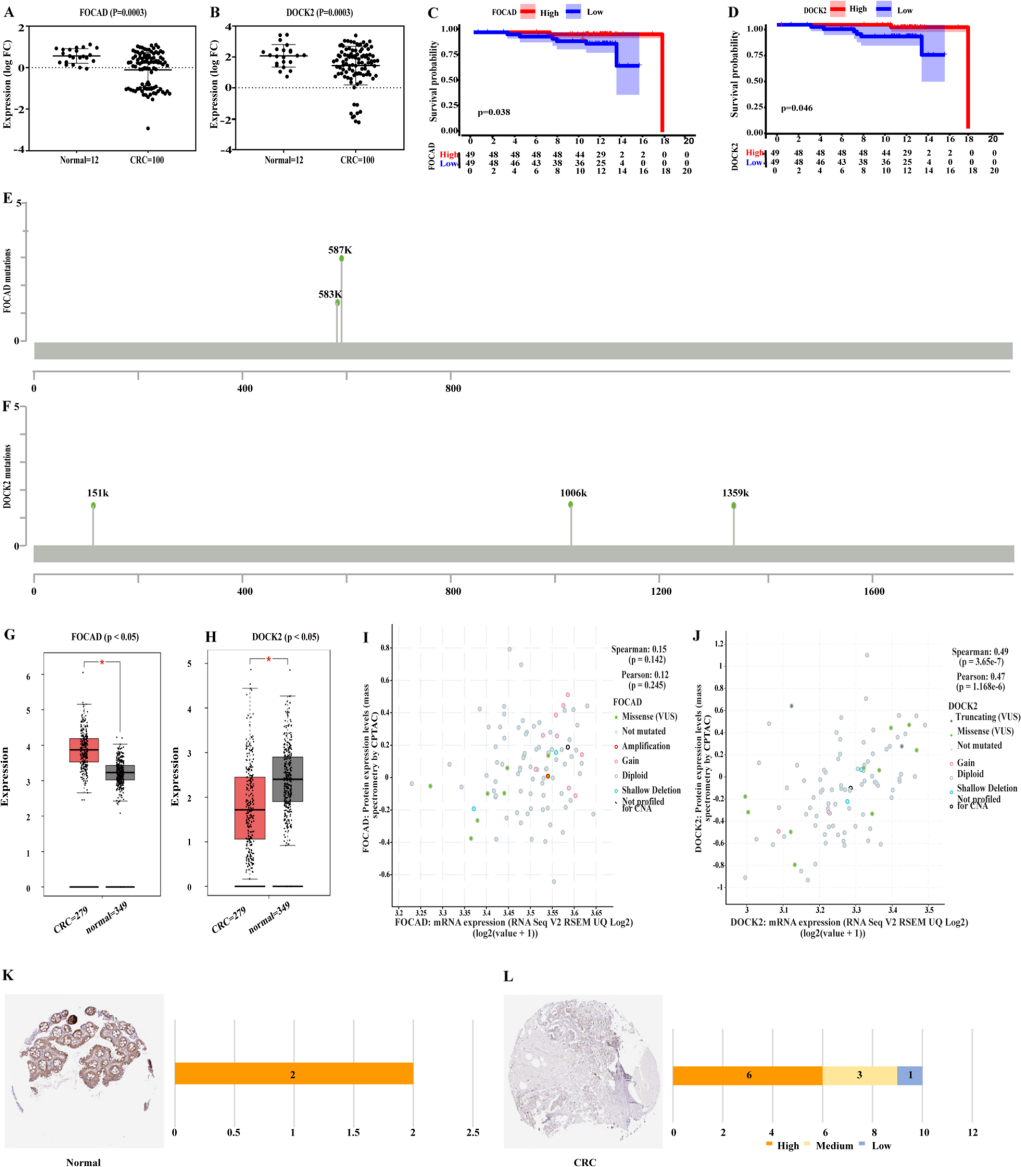
The ubiquitination of FOCAD at Lys583, Lys587 was probably relevant to the progression of CRC. (**A**–**B**) The protein expression of FOCAD and DOCK_2_ in cancerous and para-cancerous colonic tissues of CRC patients. (**C**–**D**) The survival analysis of FOCAD and DOCK_2_ using the proteomic datasets from CPTAC. (**E**–**F**) The increased ubiquitinated sites of FOCAD and DOCK_2_ in CRC cells. (**G**–**H**) The mRNA expression of FOCAD and DOCK_2_ in cancerous and para-cancerous colonic tissues of CRC patients. (**I**–**J**) The correlation between the mRNA and protein levels of FOCAD and DOCK_2_ in individuals, as analyzed using the datasets from the cbioportal database. (**K**–**L**) The protein expression of FOCAD in normal colonic tissues and CRC tissues

As shown in Fig. 4A–B, the protein expression of FOCAD and DOCK2 were both down-regulated in CRC tissues versus normal tissues (n = 100). Besides, the lower protein expression of FOCAD and DOCK2 were correlated with poor OS of CRC patients (Fig. 4C–D). Meanwhile, the ubiquitination of FOCAD at Lys583, Lys587, and DOCK2 at Lys151, Lys1006, and Lys1359 were all up-regulated in CRC cells versus normal cells (Fig. 4E–F). We considered that the decreased protein expression might be caused by the decreased mRNA content or increased ubiquitination. To clarify the protein down-regulation of FOCAD and DOCK were induced by up-regulated ubiquitination, rather than mRNA down-regulation, we analyzed the mRNA expression of FOCAD and DOCK in CRC cells versus normal cells using the RNA-Seq datasets from The Cancer Genome Atlas (TCGA). As a result, the mRNA expression of FOCAD was significantly up-regulated in CRC cells versus normal cells, while the mRNA expression of DOCK2 was significantly down-regulated in CRC cells versus normal cells (Fig. 4G–H). This result indicated that the down-regulation of the FOCAD protein was more likely to be caused by an increase of ubiquitination than DOCK2. Moreover, we analyzed the correlation between the protein level and the mRNA level of these two genes in a large cohort using data sets from the cbioportal database, and found that the DOCK2 mRNA had a higher correlation with its protein level (correlation coefficient = 0.49), while FOCAD mRNA had a lower correlation with its protein level (correlation coefficient = 0.15) (Fig. 4I–J). This further proved that FOCAD was more likely to be modified by ubiquitination to affect its protein level. Ultimately, we verified the protein expression of FOCAD using the immunohistochemical results from the HPA database and confirmed a reduced expression of FOCAD in CRC patients (Fig. 4 K–L).

### Further exploration of the role and functions of FOCAD in colon adenocarcinoma

**Fig. 5:**
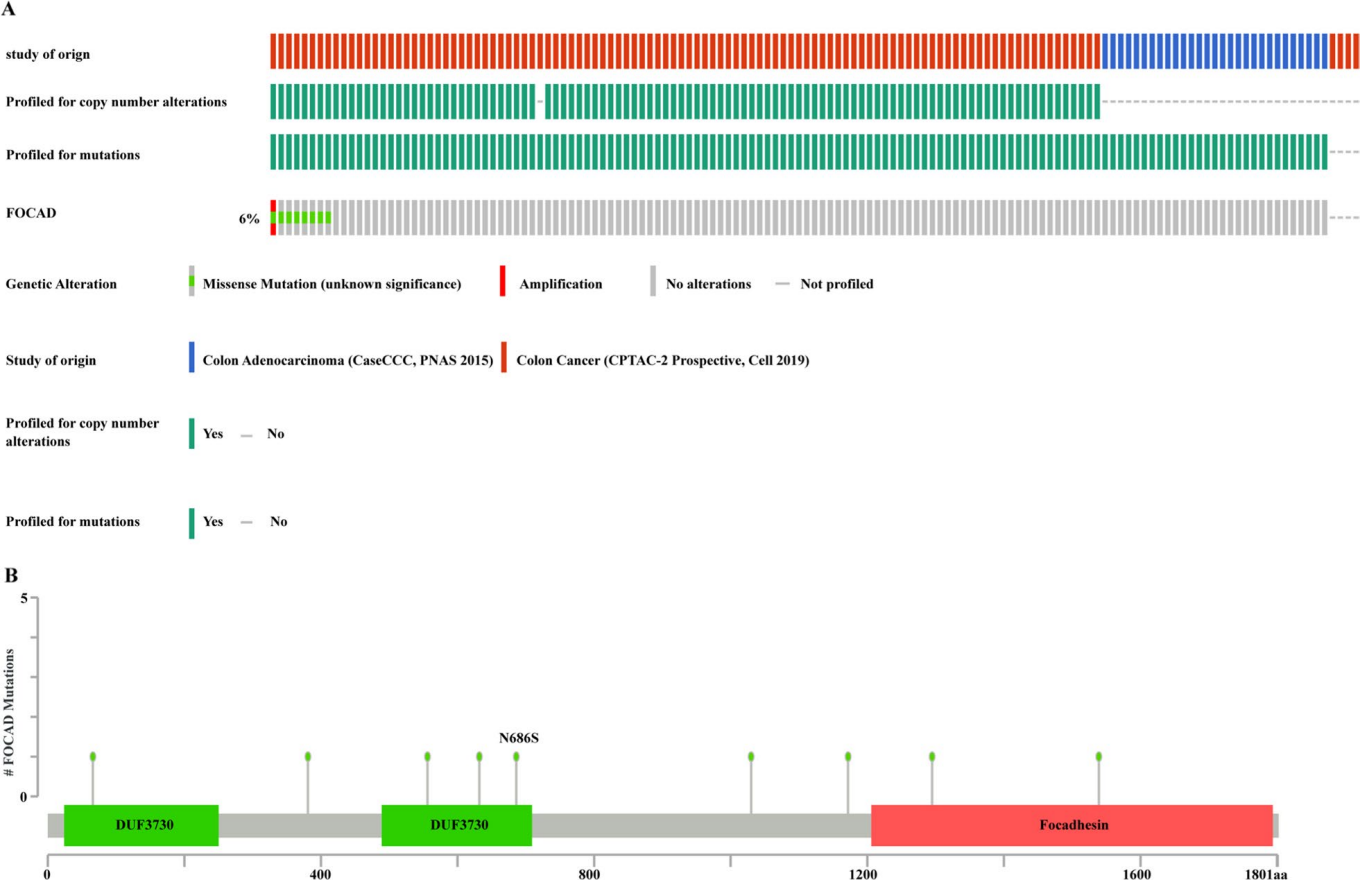
Mutation frequency and loci of FOCAD in colon adenocarcinoma. **A** Mutation frequency of FOCAD in colon adenocarcinoma. **B** Mutation sites of FOCAD in colon adenocarcinoma. These results were analyzed using the data sets from the cbioportal database.

Patients with germline deletions of FOCAD are more likely to develop intestinal polyps and tumors [12]. To further-in-depth reveal the role of FOCAD in CRC, we analyzed the mutation frequency and loci of FOCAD using the datasets from cbioportal database. The results showed that FOCAD was a frequently mutant gene (6%) in colon adenocarcinoma (Fig. 5A). Moreover, the loci of A381V, Q1179K, C66R, A1037V, R1546L, H1302P, G632C, R556Q, and N686S of FOCAD were more prone to be mutated (Fig. 5B).

**Fig. 6:**
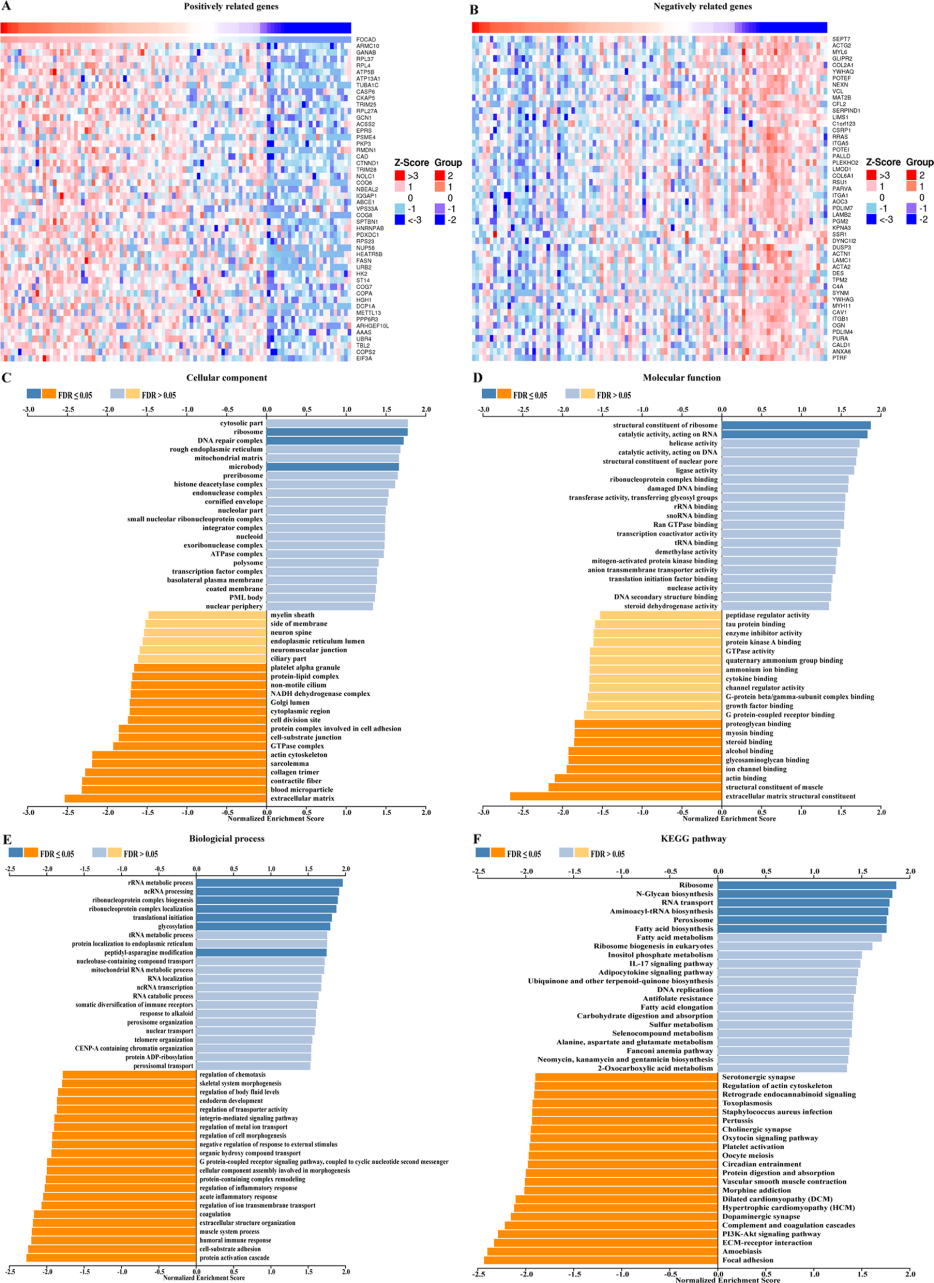
The probable functions of FOCAD in CRC. The heat maps showing the top 50 **A** positively correlated and **B** negatively correlated co-expressed proteins of FOCAD in human CRC cells. **C**–**F** GSEA analysis of the co-expressed proteins of FOCAD disclosed the potential functions of FOCAD in CRC patients

Subsequently, to further explore the functions of FOCAD in CRC, we investigated the co-expressed proteins of FOCAD in CRC patients (correlation coefficient > 0.3 or < -0.3) using the datasets from the Linkedomics database. Figure 6A, B showed the top 50 positively and negatively correlated proteins of FOCAD. Next, the Gene Set Enrichment Analysis (GSEA) disclosed that FOCAD might be associated with gene transcription and translation, PI3K-AKT pathway, immune pathways, and metabolism pathways (Fig. 6C–F)

## Discussion

Ubiquitination is a type of post-translational modification, the dyfunctions of which have been discovered in many tumors, such as breast cancer [22] and gastric cancer [23]. The disturbance of ubiquitination has also been proclaimed to be closely relevant to the initiation and progression of CRC [24]. In 2020, Zhang Y et al. uncovered the unique ubiquitination in human metastatic colon adenocarcinoma compared to the primary colon adenocarcinoma, and revealed an important role of down-regulated ubiquitination of CDK1 in carcinogenesis [25]. Distinguished from Zhang’s work, our study focused on the differentially expressed ubiquitination of CRC cells versus normal adjacent cells. Our study identified 3446 differentially expressed ubiquitination sites in CRC cells versus normal adjacent cells, providing a potentially functional context of ubiquitination for further study.

Meanwhile, we discovered 1172 proteins with up-regulated ubiquitination and 1700 proteins with down-regulated ubiquitination in CRC cells versus normal adjacent cells. As is known, biomarkers play a major role in cancer screening and diagnosing. Nowadays, carcinoembryonic antigen (CEA) and carbohydrate antigen 199 (CA199) have been widely applied in the clinic as diagnostic biomarkers for many tumors, including CRC [26]. However, these two biomarkers are not sensitive to CRC. The differentially expressed ubiquitination may be developed into molecular biomarkers in future after evaluation of the diagnostic values based on a larger cohort. Furthermore, we found that the decreased expression of FOCAD and its increased ubiquitination were potentially associated with the OS of CRC patients. Predictive biomarkers are frequently used to identify high-risk patients and predict drug responses. Until now, several potential prognostic biomarkers have been discovered [27–29], but there were still no suitable molecular biomarkers applied in the clinic. The findings of our study suggested that FOCAD and its ubiquitination might be used to predict the outcomes of CRC patients.

Next, we disclosed that the high-ubiquitinated proteins frequently participated in the regulation of tumor immune, suggesting that these ubiquitin-modifying sites may be developed into drug targets of immunotherapy. Nowadays, immune checkpoint inhibitors (ICIs) have been applied in some types of tumors, but only beneficial to patients with high microsatellite instability (dMMR-MSI-H) [30]. The most representative ICIs are the inhibitors of programmed cell death protein-1 (PD-1) /programmed death-ligand 1 (PD-L1) and cytotoxic T-lymphocyte associated antigen 4 (CTLA-4) [31–33]. Novel immunotherapeutic targets combining with known targets may increase the drug response rate.

Actually, a large number of studies have revealed that ubiquitination has a variety of functions. Except to protein degradation, ubiquitination also has non-proteolytic functions, such as receptor internalization [17], complex assembly [18], protein transporting [19], signal transduction [20], enzymatic activity modulation [21]. Consequently, the proteins with up-regulated ubiquitination are not necessarily degraded, and they may undergo changes in activity, cell location, etc. In fact, our proteomic and ubiquitinomic examination discovered 169 proteins in total had an opposite change of ubiquitination (Additional file 1: Figure S3). Some previous studies have also disclosed a similar correlation between proteome and ubiquitinome with us [34–36]. 225 proteins with up-regulated ubiquitination but unchanged protein expression were identified by Liu et al.[34]. Sap et al. found that the increasing and decreasing site-specific ubiquitination frequently occurs within the same protein and did not alter the protein levels, suggesting that the ubiquitination had non-degradative functions [35]. Another article published in 2021 demonstrated that there was no simple relationship between the peptide ubiquitination and protein abundance [36].

Moreover, we uncovered that the increased ubiquitination of FOCAD at Lys583 and Lys587 probably were survival-associated. In a previous study, FOCAD had been identified as a tumor suppressor gene in glioma, the loss of which might promote aggressiveness, enhance microtubule assembly and accelerate the G2/M phase. Besides, the decreased expression of FOCAD was relevant to worsening diffuse astrocytic gliomas’ outcome [37]. The germline deletion of FOCAD affected the opening reading frame in patients with intestinal polyp and CRC [12]. Consistently, our discoveries in this study also revealed that FOCAD was one probable tumor suppressor in CRC.

## Conclusion

To sum up, we characterized the ubiquitinome of CRC patients in this study and demonstrated the ubiquitination of FOCAD at Lys583 and Lys587 was potentially associated with the patient survival. The findings in our research provide a full view of ubiquitination in CRC patients, improve our understanding of functions of ubiquitination, and offer potential drug targets for CRC therapy.

## Supplementary Information

**Additional file 1: Figure S1.** The length of all identified ubiquitinated peptides. **Figure S2.** The numbers of all identified spectrums, peptides and proteins. **Figure S3.** The screening of potential functional ubiquitination in CRC patients.

**Additional file 2.** **Table S1**. Ubiquitination annotation. **Table S2**. Protein annotation. **Table S3**. 42 proteins having ten or more than ten ubiquitination modifications. **Table S4**. 20 ubiquitin-binding motifs. **Table S5**. 2242 proteins with an upregulated ubiquitination and 1204 proteins with a down-regulated ubiquitination. **Table S6**. 1172 up-regulated proteins and 1700 down-regulated proteins. **Table S7**. 646 Secretory proteins. **Table S8**. 53 proteins having an upregulated expression simultaneously with a down-regulated ubiquitination and 116 proteins having down-regulated expression simultaneously with an up-regulated ubiquitination. **Table S9**. 7 proteins having an up-regulated expression simultaneously with a down-regulated ubiquitination. **Table S10**. 52 proteins having a down-regulated expression simultaneously with an up-regulated ubiquitination. **Table S11**. 101 proteins which were probably associated with the overall survival rates of CRC patients.

## Data Availability

The mass spectrometry proteomics data have been unloaded and deposited onto the ProteomeXchange Consortium through the PRIDE partner repository: PXD028504.

## Abbreviations

CRC: Colorectal cancer
CPTAC: The national cancer institute’s clinical proteomic tumor analysis consortium
GEPIA: The gene expression profiling interactive analysis
HPA: The human protein altas
MS: Mass-spectrometry
FC: Fold change
FDR: False discovery rate
GO: Gene ontology
KEGG: Kyoto encyclopedia of genes and genomes
PASEF: Parallel accumulation serial fragmentation
TCGA: The cancer genome atlas program
OS: Overall survival rate
