# Supplementary material for: Comprehensive characterization of ubiquitinome of human colorectal cancer and identification of potential survival-related ubiquitination
Source: J Transl Med. 2022 Oct 2;20:445. doi: 10.1186/s12967-022-03645-8 (PMC9528151; doi:10.1186/s12967-022-03645-8)
Supplement: Supplementary file 1 — Additional file 1: Figure S1. The length of all identified ubiquitinated peptides. Figure S2. The numbers of all identified spectrums, peptides and proteins. Figure S3. The screening of potential functional ubiquitination in CRC patients. [file 12967_2022_3645_MOESM1_ESM.docx]

**Title page**

**Title:** Comprehensive characterization of ubiquitinome of human colorectal cancer and identification of potential survival-related ubiquitination

**Running title**: Ubiquintome of human colorectal cancer

**Authors and affiliations: Yan** **Yang****^1,2*^, Wei Zhang^1, 3*^,** **Liewen Lin^3^,** **Jingquan He^3^, Jingjing Dong^1,2^, Bin Yan^3^, Wanxia Cai^3^, Yumei Chen^3^,** **Lianghong Yin^1,2^, Donge Tang^3#^,** **Fanna Liu^1,2#^, Yong Dai ^3#^**

**Author details:**

^1^ The First Affiliated Hospital, Jinan University, Guangzhou 510632, China.

^2^ Department of Nephrology, Institute of Nephrology and Blood Purification, the First Affiliated Hospital of Jinan University, Jinan University, Guangzhou 510632, China.

^3^ Department of Clinical Medical Research Center, The Second Clinical Medical College, Jinan University (Shenzhen People's Hospital), Shenzhen 518020, China.

**#Corresponding authors:**

Donge Tang, Department of Clinical Medical Research Center, Guangdong Provincial Engineering Research Center of Autoimmune Disease Precision Medicine, The Second Clinical Medical College, Jinan University (Shenzhen People's Hospital), Shenzhen 518020, China. E-mail address: [donge66@126.com](mailto:donge66@126.com); Fanna Liu, Department of Nephrology, The First Affiliated Hospital of Jinan University, 613 W. Huangpu Avenue, Guangzhou, Guangdong 510632, China. E-mail address: tliufana@jnu.edu.cn; or Yong Dai, Department of Clinical Medical Research Center, Guangdong Provincial Engineering Research Center of Autoimmune Disease Precision Medicine, The Second Clinical Medical College, Jinan University (Shenzhen People's Hospital), Shenzhen 518020, China. Tel/Fax: (86) 0755-22942780. E-mail address: [daiyong22@aliyun.com](mailto:daiyong22@aliyun.com) or dai.yong@szhospital.com.

Co-Authors: These authors contributed equally to this work.

**Keywords****: Ubiquintome, multi-omics study, colorectal cancer, FOCAD, DOCK2**

**Supplementary Figure S1**


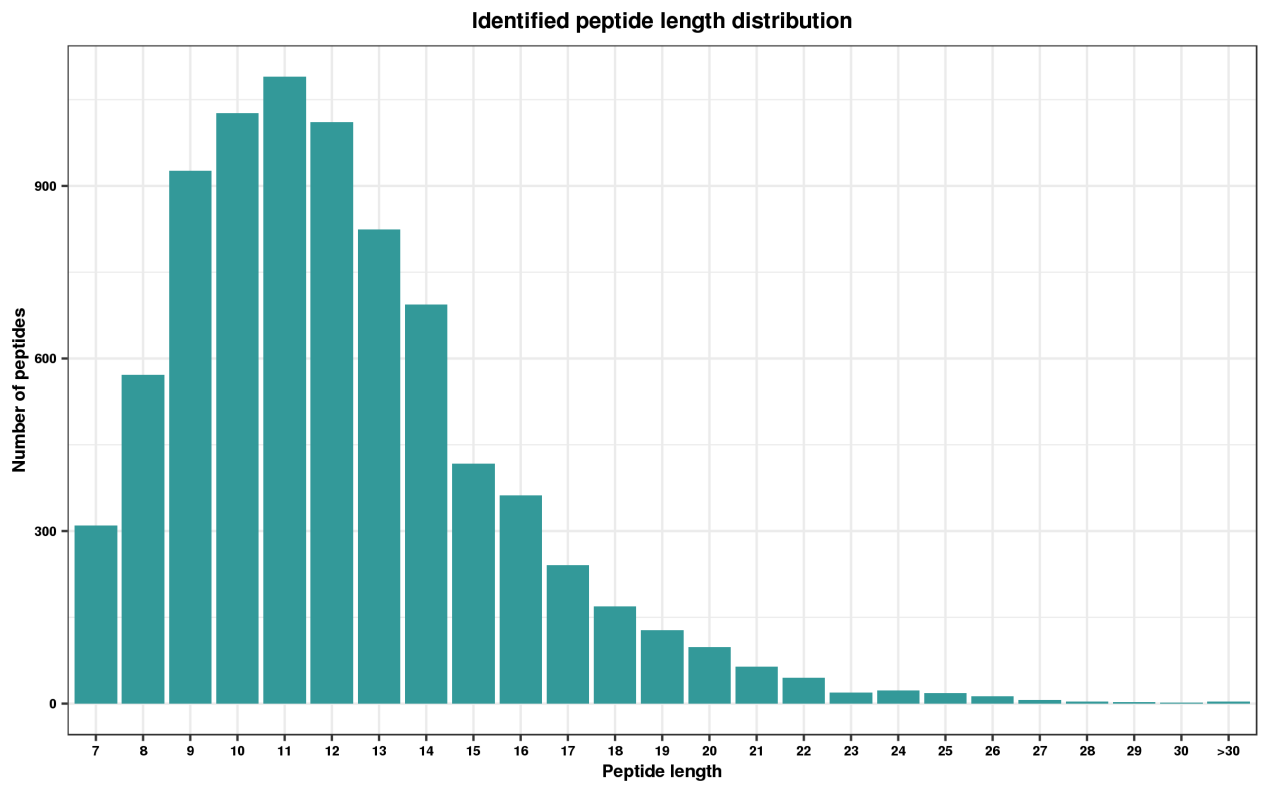


**The length of all identified ubiquitinated peptides.**

**Supplementary Figure S2**


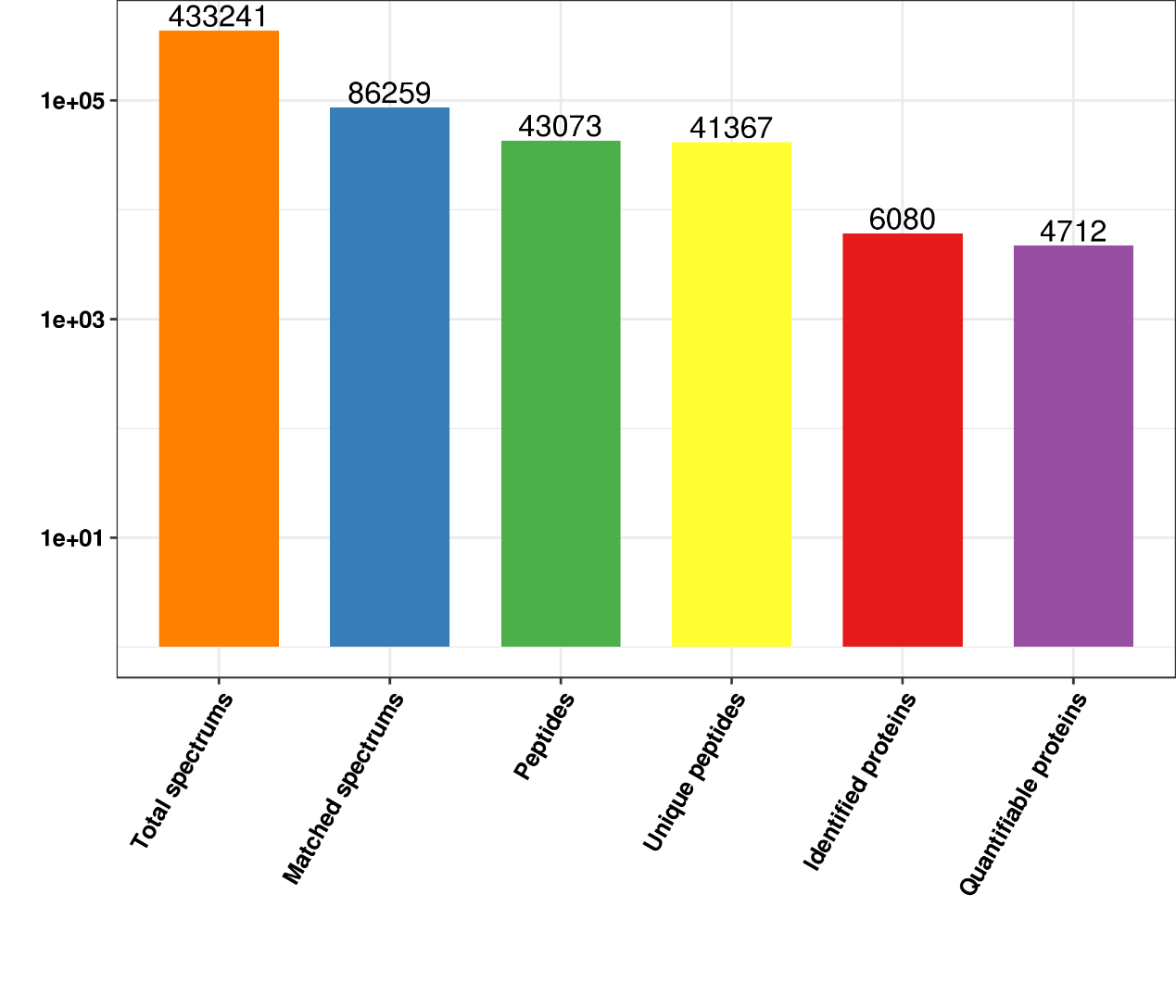
 **The numbers of all identified spectrums, peptides and proteins of** **proteomic examination**


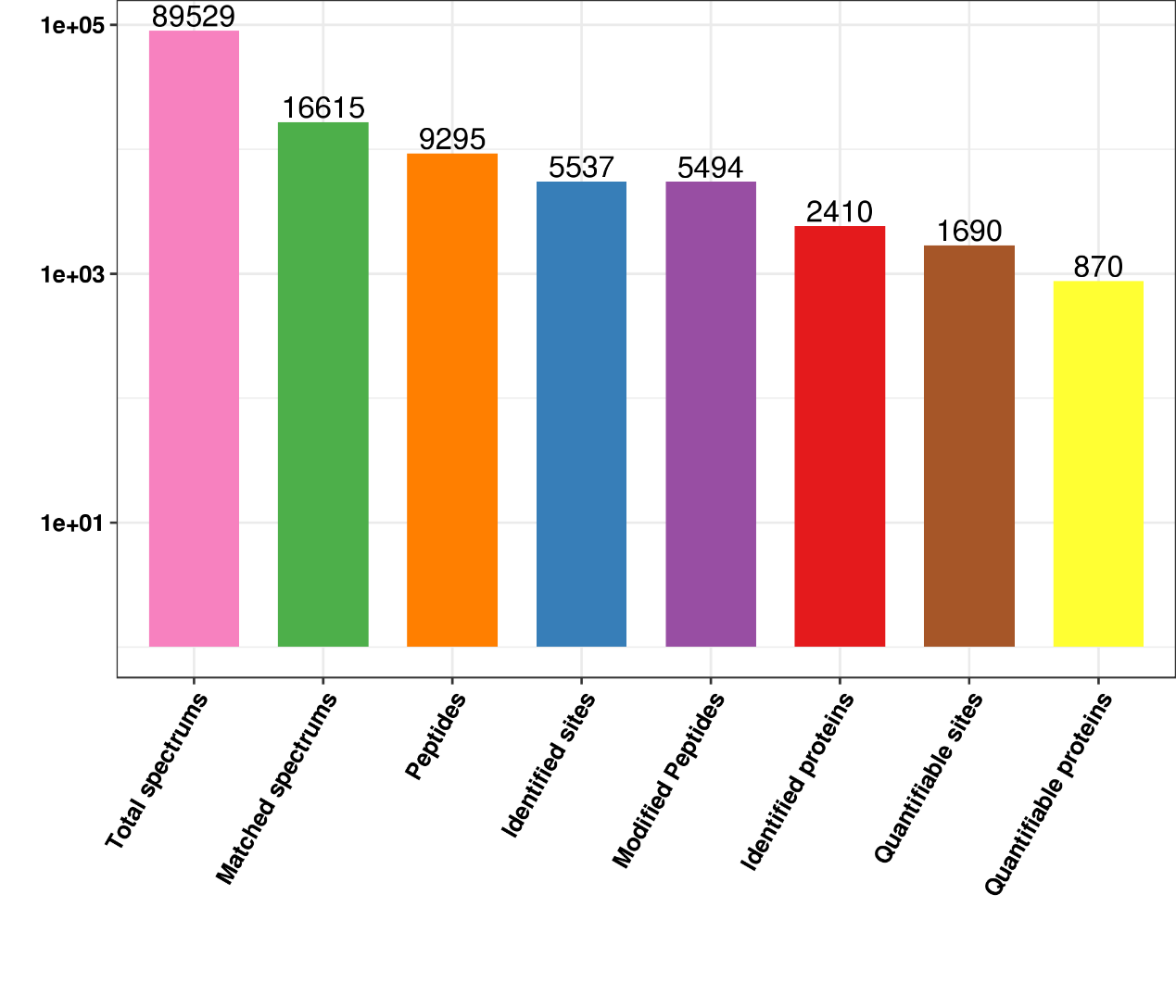
**The numbers of all identified spectrums, peptides and proteins of ubiquitinomic examination.**

**Supplementary Figure S3**


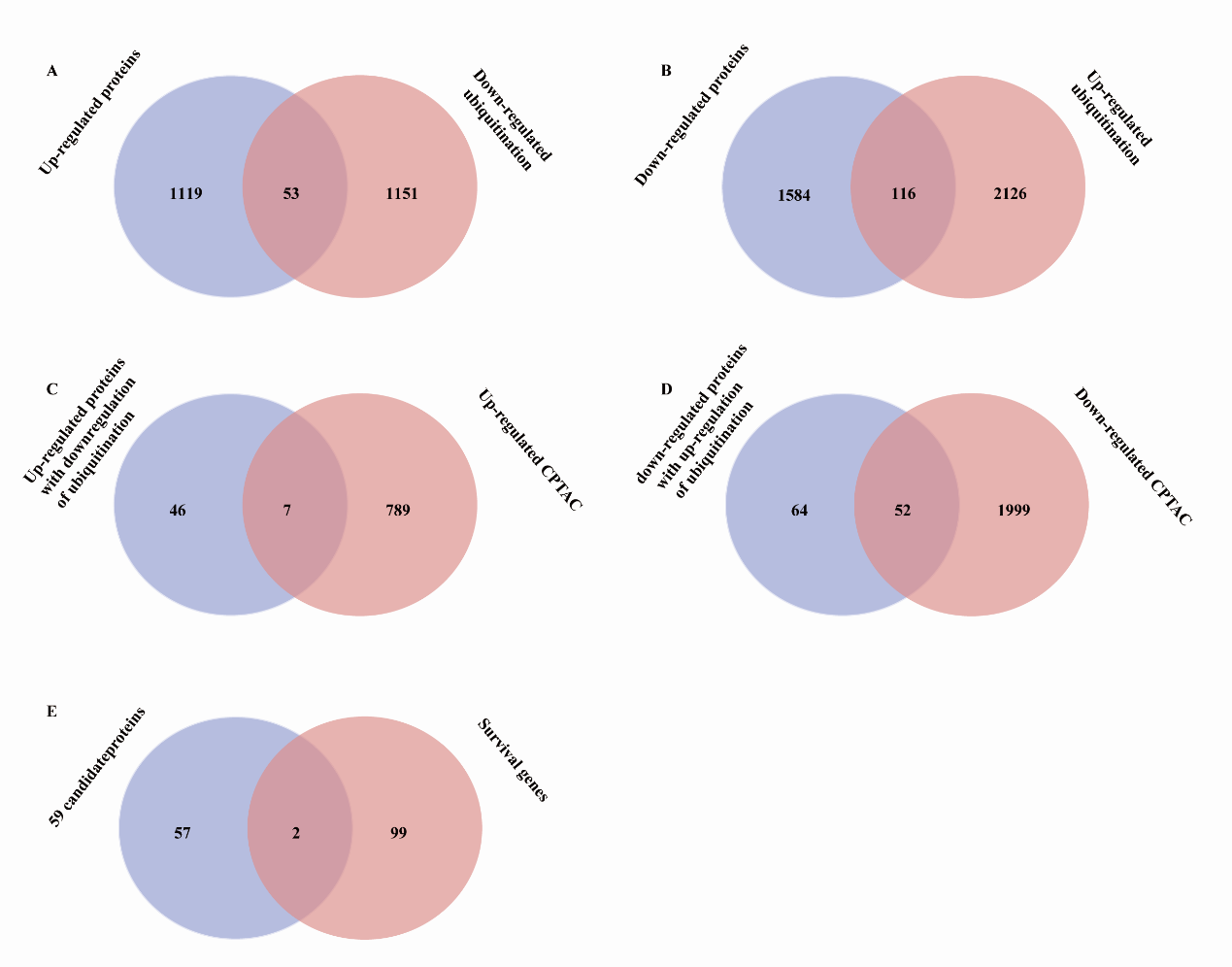


**The screening of potential functional ubiquitination in CRC patients.**

(A) The overlapping analysis of 1172 up-regulated proteins and 1204 proteins with down-regulated ubiquitination. (B) The overlapping analysis of 1700 down-regulated proteins and 2242 proteins with up-regulated ubiquitination. (C) 53 up-regulated proteins identified by our proteomic datasets intersected with 796 up-regulated proteins the datasets of CPTAC. (D) 116 down-regulated proteins identified by our proteomic datasets intersected with 2051 down-regulated proteins the datasets of CPTAC. (E) The intersection analysis of 59 ubiquitin-modified proteins identified in Figures 5C, D, and 101 OS-relevant proteins.
